# Growth and characterization of Cu(In,Ga)Se_2_ thin films by nanosecond and femtosecond pulsed laser deposition

**DOI:** 10.1186/1556-276X-9-280

**Published:** 2014-06-02

**Authors:** Shih-Chen Chen, Dan-Hua Hsieh, Hsin Jiang, Yu-Kuang Liao, Fang-I Lai, Chyong-Hua Chen, Chih Wei Luo, Jenh-Yih Juang, Yu-Lun Chueh, Kaung-Hsiung Wu, Hao-Chung Kuo

**Affiliations:** 1 Department of Photonics and Institute of Electro-Optical Engineering, National Chiao-Tung University, Hsinchu 30010, Taiwan; 2 Department of Electrophysics, National Chiao-Tung University, Hsinchu 30010, Taiwan; 3 Department of Materials Science and Engineering, National Tsing Hua University, Hsinchu 30013, Taiwan; 4 Department of Photonic Engineering, Yuan-Ze University, Taoyuan 32003, Taiwan

**Keywords:** CIGS, Pulsed laser deposition, Femtosecond, Photoluminescence, Pump probe

## Abstract

In this work, CuIn_1 - *x*_Ga_*x*_Se_2_ (CIGS) thin films were prepared by nanosecond (ns)- and femtosecond (fs)-pulsed laser deposition (PLD) processes. Different film growth mechanisms were discussed in perspective of the laser-produced plasmas and crystal structures. The fs-PLD has successfully improved the inherent flaws, Cu_2 - *x*_Se, and air voids ubiquitously observed in ns-PLD-derived CIGS thin films. Moreover, the prominent antireflection and excellent crystalline structures were obtained in the fs-PLD-derived CIGS thin films. The absorption spectra suggest the divergence in energy levels of radiative defects brought by the inhomogeneous distribution of elements in the fs-PLD CIGS, which has also been supported by comparing photoluminescence (PL) spectra of ns- and fs-PLD CIGS thin films at 15 K. Finally, the superior carrier transport properties in fs-PLD CIGS were confirmed by fs pump-probe spectroscopy and four-probe measurements. The present results indicate a promising way for preparing high-quality CIGS thin films via fs-PLD.

## Background

CuIn_1 - *x*_Ga_*x*_Se_2_ (CIGS) has been extensively regarded as the most favorable absorber layer for thin film photovoltaic devices. CIGS possesses superior absorption characteristics due to its direct bandgap, which can be engineered by the partial substitution of indium by gallium atoms. Recently, the reported thin film CIGS-based solar cells have achieved the highest efficiency of 20.8% among all thin film solar cells at laboratory level [1]. The absorber layers for high-performance CIGS-based solar cells are usually prepared by vacuum processes (such as co-evaporation or sputtering). However, post-selenization and precise control of deposition parameters are required in both vacuum approaches [2,3]. In contrast, pulsed laser deposition (PLD) is an alternative way that possesses the advantages of simple usage and good transfer of stoichiometry of target composition without post-selenization [4,5]. All of these advantages are beneficial to obtain high-quality and reproducible CIGS thin films at low cost and are also suitable for investigating the underlying physical mechanisms that limit the efficiency.

The first PLD CIGS thin films were reported by Kusmartseva et al.; they investigated the effects of growth temperature and substrate material on the films [5]. Subsequently, extensive efforts have been devoted to study the correlations between the properties of PLD CIGS thin films and their growth parameters (such as composition of target, target-substrate distance, annealing time, laser repetition rate, and energy fluence) [6-9]. The laser sources used in the early-stage research were excimer lasers with pulse duration of tens of nanosecond (ns). Recently, Tsai et al. deposited CIGS thin films utilizing the femtosecond (fs) mode-locked Ti:sapphire laser rather than the excimer laser [10]. The annealing effects on the crystallization, microstructure, surface composition, and photoelectrical property of CIGS films were reported.

Comparisons of the growth mechanisms of PLD processes with different laser sources are crucial to understand the characteristics of high-quality CIGS thin films. However, the effect of laser sources on characteristics and growth mechanism of PLD CIGS thin films has not been investigated yet. In this study, the PLD CIGS thin films prepared by ns excimer laser and fs Ti:sapphire laser are compared to delineate the intimate correlations between the film growth mechanism and the associated plasma dynamics. Surface morphologies and grain structures were examined by scanning electron microscope (SEM). The crystalline structures and elemental distributions were analyzed by X-ray diffraction (XRD) spectrum and energy dispersive spectrum (EDS). The reflectance of the PLD CIGS films was measured by UV-visible-near infrared (NIR) spectrophotometer. In addition, defects and their effect on carrier dynamics were measured and investigated by photoluminescence (PL) and fs pump-probe spectroscopy.

## Methods

### Growth of PLD CIGS thin films

The laser sources used in this experiment were KrF excimer laser (wavelength = 248 nm, pulse width = 20 ns, fluence approximately 10 J/cm^2^, pulse repetition rate = 10 Hz) and Ti:sapphire mode-locked laser (wavelength = 800 nm, pulse width = 100 fs, fluence approximately 1 J/cm^2^, pulse repetition rate = 5,000 Hz), which were for ns-PLD and fs-PLD growth, respectively. The CIGS thin films were deposited on soda-lime glass (SLG) substrates in a vacuum chamber. The background pressure was kept at 4 × 10^-6^ Torr. The distance between the target and substrate was 4 cm. The substrate temperature was monitored by a thermocouple attached to the substrate holder and was kept at optimal temperature of 500°C during the deposition processes.

### Characterization

Powder XRD was conducted by a Bruker D2 PHASER X-ray spectrometer (Ettlingen, Germany) under irradiation of mono-chrome Cu-K_α_ (*λ* approximately 1.54 Å). The morphology, nanostructure, and elemental compositions of chalcopyrite films were obtained by field emission SEM (JSE-7001, JEOL, Tokyo, Japan) with attached accessory of EDS (INCA analysis system, Oxford Instruments, Oxfordshire, UK). Reflectance spectroscopy was acquired by the spectrophotometer (U-4100 UV-visible-NIR spectrophotometer, Hitachi, Tokyo, Japan). PL measurements were carried out using a 635-nm CW diode laser excitation source. The signal was dispersed by a monochromator and detected by an InGaAs photodiode (working range = 900 to 2,100 nm). A continuous helium cryostat was used for low-temperature PL measurements. For pump-probe measurements, a commercial Ti:sapphire laser system providing short pulses (approximately 30 fs) with repetition rate of 75 MHz and wavelength of 800 nm (*hv* = 1.55 eV) was used. The pump beam was focused at a diameter of about 50 μm with pump fluence ranging from 15.2 to 45.7 μJ/cm^2^, while the probe fluence was fixed at approximately 1 μJ/cm^2^ at spot diameter of 20 μm. The pump pulses were modulated at 2 KHz with a chopper. A mechanical delay stage was used to vary the time delay between the pump and probe pulses. The transient reflectivity change Δ*R*/*R* of the probe beam was measured as a function of the pump-probe delay time. The small reflected signals were detected and fed into a lock-in amplifier.

## Results and discussion

**Figure 1:**
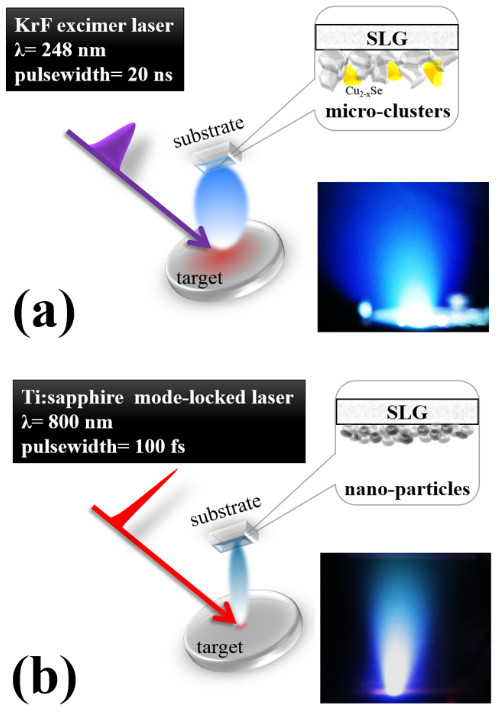
**Schematic illustrations and photos of laser-produced plasmas on CIGS target. (a)** ns-PLD and **(b)** fs-PLD.

Figure 1a,b shows the laser-produced plasmas (LPP) at the surface of the CIGS target by ns and fs laser, respectively. It exhibits substantial dissimilarities in LPPs that can be explained by the various laser-target interactions. For the ns-PLD process (Figure 1a), there is much residual heat, which is caused by the longer duration of laser pulse, as the pulse laser hits the target. The residual heat is due to the picosecond order of both the heat conduction time and ion energy transfer time, which is much faster than the pulse width of the excimer laser. It leads to the mixing of the melted CIGS (gray color) debris with the direct-transferred undesirable Cu_2_Se secondary phases (yellow color) from the target as clusters were ejected along with the plasma in expansive directions. The effect of residual heat can spread to a wider range in the target, thus leading to an enlarged heat-affected zone (HAZ) (red region) that brings the plasma and debris with variation in energy and random transportation directions. This is why the expansive plasma was observed as shown in the inset of Figure 1a. Nonetheless, these large clusters can re-crystallize into a preferred orientation directed by the flow of the remaining residual energy of the laser pulses and the thermal energy from the heated substrate.

On the contrary, the highly localized interactions with target minimize the HAZ by the fs pulse laser. This is because the duration of laser pulse is shorter than the heat conduction time, so the residual energy can be eliminated. The main mechanism of producing plasma by fs pulse laser is coulomb explosion, a process that ionizes atoms in a solid-state target through an extremely intensive electric field, rather than conventional evaporation. With the absence of residual heat, concentrated plasma was generated by fs laser pulses (Figure 1b), which consists of the mixture of atoms and nanometer particles. These ingredients tend to construct the same crystal structure of the polycrystalline target when reaching the heated substrate due to the non-participation of residual heat as the re-crystallization energy. Similar properties of the fs pulse-induced laser plume were discussed by Verhoff et al [11].

**Figure 2:**
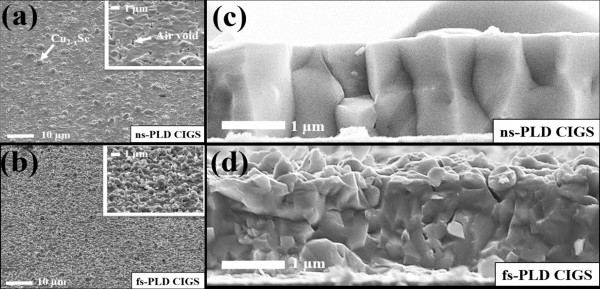
**SEM images of ns-PLD CIGS and fs-PLD CIGS.** Top-view SEM images of **(a)** ns-PLD CIGS and **(b)** fs-PLD CIGS. Side-view SEM images of **(c)** ns-PLD CIGS and **(d)** fs-PLD CIGS.

Figure 2a,b shows the surface and grain morphologies of both ns-PLD and fs-PLD CIGS thin films. CIGS film deposited by the ns-PLD growth was found to have smooth surface and larger grain size, while much rougher surface with smaller grains was observed in films deposited by the fs-PLD growth. Figure 2c shows the side-view SEM image of the ns-PLD CIGS thin film, in which the grain boundaries (GBs) can be clearly observed. In contrast, the GBs of the fs-PLD CIGS thin film are barely seen as shown in Figure 2d, which indicates a more compact structure as expected. As shown in Figure 2a, there are a lot of micro-clusters generated due to the residual heat generated by ns laser pulses. It has also been found that the secondary phases (Cu_2 - *x*_Se) with Cu/In/Ga/Se = 62.92:1.42:0.82:34.84 characterized by EDS tend to segregate on the surface and appear as large droplets indicated by the white arrow shown in Figure 2a [9]. However, it is evident from Figure 2b that the segregation of secondary phases is significantly reduced in films obtained by fs-PLD [11]. Moreover, air voids occurring at grain boundaries (marked by the white arrow in the inset of Figure 1a) were observed in films deposited by the ns-PLD. The formation of air voids between grains is most likely due to the stack of the larger clusters and debris. It is worthy to note that both of the abovementioned microstructure features exhibited in films deposited by the ns-PLD can lead to substantial current leakage in devices. Such detrimental disadvantages, nevertheless, can be successfully removed with a concentrated and oriented plume consisting of atoms and nanometer-cluster mixtures resulting from the localized strong electric field ionization on the target by using the fs pulses [12]. In addition, ingredients of the nanometer-cluster mixture evidently resulted in a much more compact CIGS films (Figure 2b). Consequently, the inherent nanostructure uniformly distributed on the surface of fs-PLD-derived CIGS film is observed instead of the micrometer-sized droplets of the secondary phases.

**Figure 3:**
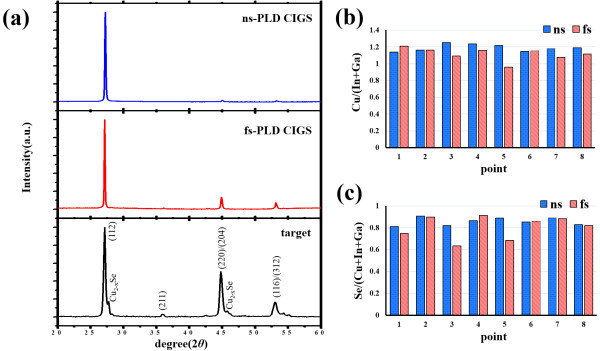
**Material characterizations of target and both PLD films. (a)** XRD spectra, **(b)** CIG ratio, and **(c)** SCIG ratio for both PLD films.

The XRD patterns of the CIGS target and the two CIGS thin films are presented in Figure 3a. In the pattern of the CIGS target, the main peaks are broadened and degenerated to the peaks of binary crystals of Cu_2 - *x*_Se_*x*_, which is commonly found in the hot-pressed CIGS pellet. In contrast, the homogeneous phase and remarkable crystallinity can be found in the two CIGS thin films. The polycrystalline feature with the chalcopyrite structure in the CIGS target is directly transferred to the CIGS films obtained by both ns- and fs-PLD processes. In addition, the full-width at half maximum (FWHM) of (112) diffraction peaks are 0.18° and 0.14° in ns-PLD and fs-PLD CIGS thin films, respectively. The smaller FWHM is indicative of larger grain size and better crystallinity in the fs-PLD CIGS. Furthermore, the existence of the (220)-oriented peak, which is beneficial for reducing the surface recombination of the CIGS absorber layer due to higher work function, is largely preserved only in films grown by the fs-PLD [13]. Preliminary studies have also shown that the relaxed structure usually accompanies with the broadened peak of (112) orientation, which is associated with high degree of structural disorder [14]. The high degree of structural disorder can be successfully suppressed for the fs-PLD CIGS thin film because of the well-crystalline characteristics confirmed by XRD spectra. The analyses of elemental composition ratios of CIG ([Cu]/[In] + [Ga]) and SCIG ([Se]/[Cu] + [In] + [Ga]) were carried out using the EDS measurements as shown in Figure 3b,c, respectively, where we randomly selected eight points from both PLD films for statistical analysis. It is observed that the ns-PLD CIGS film has more homogenous elemental distribution and is most likely due to the (112) dominant phase. Furthermore, compositions of copper and selenium of the ns-PLD CIGS film are averagely higher than that of the fs-PLD CIGS film. Other studies have reported the existence of more selenium deficiencies in PLD CIGS films [15]. This non-stoichiometry is more significant in the fs-PLD CIGS. These results could be related to the high vapor pressure of selenium. When the target is under the fs laser irradiation, the atom and nanoparticle mixture is evaporated by ultrashort pulses. During the flight of the mixture to the substrate, ‘re-evaporation’ of the nanoparticles happens and selectively decreases the elements in the mixture due to the insufficient energy that maintains the flight of the mixture to the substrate. The results agree with the fact that the pulse energy of the fs laser is much smaller than that of the ns laser (the pulse energy is 0.2 and 400 mJ for fs-PLD and ns-PLD, respectively). Re-evaporation can be significantly more effective in the mixture obtained by the fs laser pulses, which is of atomic and nanoparticle scale [14]. On the other hand, the secondary phase (Cu_2 - *x*_Se) clusters were ‘ablated’ from the target in the ns-PLD at its pristine phase (therefore, less re-evaporation can cause element loss). Moreover, the binary crystals also give rise to higher concentrations of copper and selenium in the thin film.

**Figure 4:**
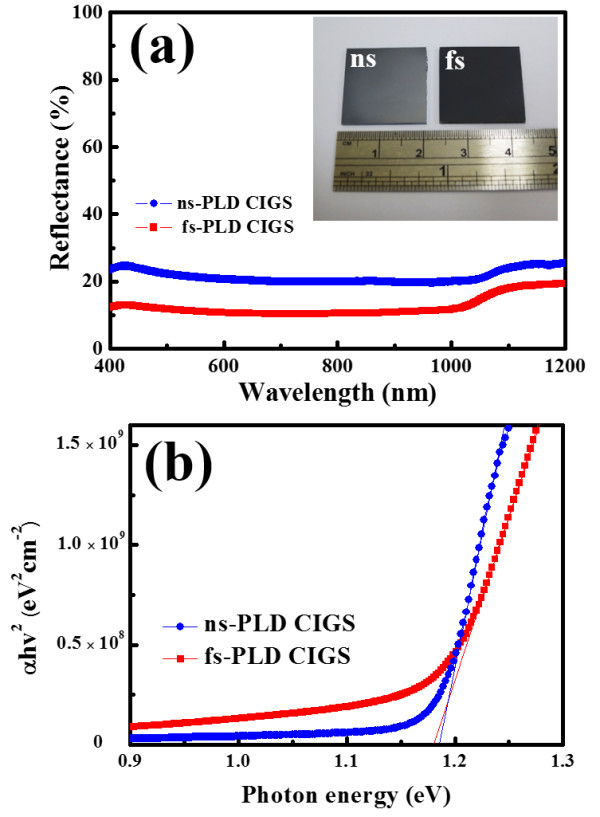
**Reflectance (a) and absorption (b) spectra of ns- and fs-PLD CIGS thin films.** The inset in (a) shows the photo of the two CIGS thin films.

The reflectance of the PLD CIGS thin films were measured as shown in Figure 4a. Obviously, the reduced reflectance is achieved in the fs-PLD CIGS film, as compared with that of the ns-PLD film. The reduced reflectance of the fs-PLD CIGS film can be attributed to the inherent nanostructure on the surface during the PLD growth process. The significant contrast in color also reveals the anti-reflection effect of the fs-PLD CIGS thin film, as shown in the inset of Figure 4a. It is a prominent property compared to the nanostructured CIGS film prepared by an extra etching process [16]. In addition, the ns- and fs-PLD CIGS thin films have a similar bandgap value of approximately 1.2 eV extracted from absorption spectra, as shown in Figure 4b. The value is well consistent with the bandgap of the target with elemental compositions of Cu/In/Ga/Se = 1:0.7:0.3:2, respectively, revealing that the variation in elementary compositions in the fs-PLD CIGS (Figure 3b,c) is localized, while the global composition of the film still remained unchanged with the same composition as that of the target. Furthermore, fs-PLD CIGS shows a longer absorption tail due to the more diverged sub-band gap energy levels of radiative defects, which is most likely resulted from the local inhomogeneous distributions of elements.

**Figure 5:**
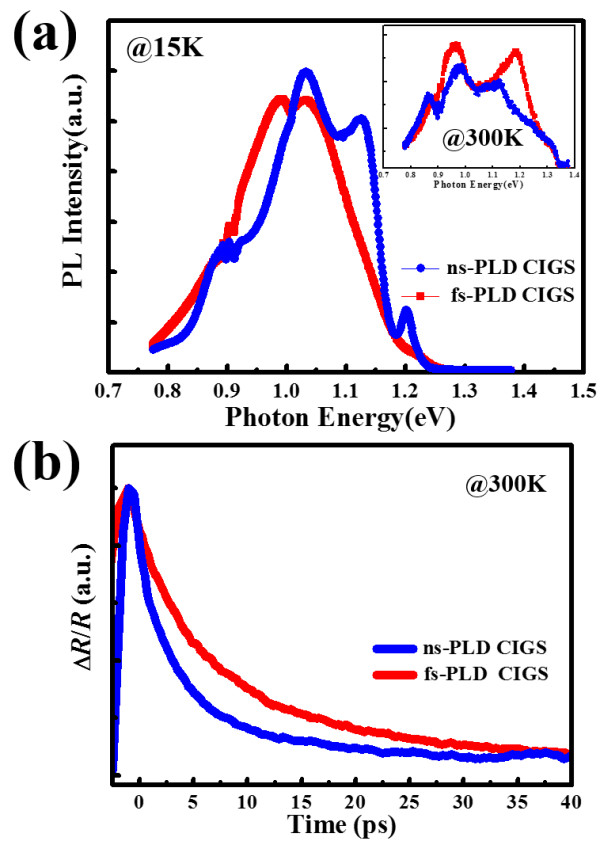
PL spectra (a) and fs pump-probe spectra (b) for ns-PLD (blue) and fs-PLD (red) CIGS thin films.

Many studies have suggested that the defects of CIGS thin films are crucial to the performance of their device performances. PL is a powerful tool to shed light on defects arising from the deviation of stoichiometry [17]. Figure 5a shows the PL spectra of fs- and ns-PLD CIGS thin films at 15 K and room temperature (see the inset) without normalization, in which PL peaks at 1.2 eV for ns-PLD CIGS agrees well with the bandgap value obtained from the absorption spectrum (Figure 4b). Hence, we assign this peak as band-to-band transition, and other PL emission peaks with energy lower than 1.2 eV are assigned to different radiative defect-related transitions. At 15 K, where transitions between the defect levels are the dominant processes for CIGS, the intensity of the two PL spectra is comparable, suggesting that the defect type and concentration in the two samples are similar. By comparison, it can be seen that individual PL emission peaks can only be resolved from the PL spectrum of the ns-PLD CIGS, while no discrete PL emission peaks can be observed from that of the fs-PLD CIGS thin film. This could be due to the fluctuations of defect energy levels in the fs-PLD CIGS thin film, which broadens the FWHM of the PL emission peaks associated with all radiative defect-related transitions. The increased overlapping of the PL emission peaks, in turn, results in the unresolvable spectrum. Such fluctuations in radiative defect energy levels have also been observed in the absorption spectrum of the fs-PLD CIGS thin film shown in Figure 4b. The absorption spectrum of the fs-PLD CIGS shows a tail at energies below its bandgap energy of 1.2 eV, indicating that energy level fluctuations related to the sub-band gap radiative defects might have been responsible for the photon absorptions observed at energies smaller than the intrinsic CIGS bandgap energy. The absorption tail can also be observed in the absorption spectrum of the ns-PLD CIGS thin film. Yet, the tail is much less significant for the ns-PLD CIGS film, presumably due to the fact that the individual radiative defect energy levels in ns-PLD CIGS film are more concentrated and less fluctuating. The discreteness of the PL emission peaks seen in the PL spectrum of the ns-PLD CIGS films evidently lends strong support to the above conjecture. At room temperature, the ns-PLD CIGS film shows a weaker PL intensity than that of the fs-PLD CIGS, which is due to the higher concentration of non-radiative recombination centers induced by surface state between CIGS/Cu_2 - *x*_Se and CIGS/void interfaces. In addition, the stronger PL intensity of the fs-PLD CIGS can correspond to the existence of the (220)-oriented peak whose higher work function is beneficial for reducing the surface recombination. The results indicate that the fs-PLD CIGS film is much more promising for device performance compared to the ns-PLD CIGS film.

The defects in the CIGS thin films can also affect the carrier dynamics, hence their device performance. To this respect, carrier dynamics in CIGS thin films obtained by different PLD processes were investigated by fs pump-probe spectroscopy, which is a technique ubiquitously adopted to delineate the non-equilibrium carrier dynamics in semiconductors [18,19]. Figure 5b shows the reflectivity transient in both films with a pumping power of 30.4 μJ/cm^2^ at room temperature. It is apparent from Figure 5b that the carrier lifetime is much longer in the fs-PLD CIGS film. The defect-related non-radiative recombination lifetime (*τ*_n_) can be derived from the results obtained by using different pumping fluences. It showed that the *τ*_n_ of ns- and fs-PLD CIGS films are 20 and 30 ps, respectively, revealing that the Shockley-Read-Hall (SRH) mechanism is more dominant in the ns-PLD CIGS at room temperature because of the existence of CIGS/Cu_2 - *x*_Se and CIGS/void interfaces. On the other hand, the longer lifetime in the fs-PLD CIGS suggests less SRH recombination that is consistent with the existence of the (220) orientation. Finally, we examined the electrical properties by van der Pauw four-probe measurements. The resistivity values of ns- and fs-PLD CIGS thin films were approximately 66.0 Ω cm and approximately 0.1 Ω cm, respectively. The higher resistivity of the ns-PLD CIGS thin films can be attributed to the higher concentration of non-radiative recombination center verified by PL and pump-probe measurements. The superior carrier transport properties exhibited in the fs-PLD CIGS film again could be attributed to the substantial improvements realized in suppressing the formation of Cu_2 - *x*_Se secondary phase and air voids by the fs-PLD process.

## Conclusions

In summary, comparative investigations on the structure-property relations of CIGS thin films prepared by ns- and fs-PLD processes were conducted. The residual heat remaining on the target due to pulse duration difference was found to result in drastically different appearance of the laser-produced plasmas; hence, it led to vastly different film growth mechanisms and eventual film microstructures. The CIGS thin film prepared by fs-PLD, as compared to that obtained by the ns-PLD process, evidently exhibits much better film quality and superior carrier transport properties, primarily due to the removal of Cu_2 - *x*_Se and air voids. In addition, the fs-PLD CIGS thin film also exhibits significantly better antireflection characteristic over a wavelength range of 400 to 1,200 nm. The absorption spectra suggest the divergence in energy levels of radiative defects brought by the inhomogeneous distribution of elements in fs-PLD CIGS. Such inference is strongly supported by comparing the PL spectra between the ns- and fs-PLD CIGS thin films at 15 K. Room temperature PL spectra of ns- and fs-PLD CIGS thin films suggest that in the ns-PLD CIGS films, there might exist more surface states at CIGS/Cu_2 - *x*_Se and CIGS/void interfaces, which may act as the non-radiative recombination centers. Finally, fs pump-probe spectroscopy and four-probe measurements reveal that the fs-PLD CIGS films have a much longer carrier lifetime and significantly lower resistivity, both are beneficial for photovoltaic applications. The present results convincingly indicate that the fs-PLD process is a very promising method for preparing high-quality CIGS thin films.

## Abbreviations

CIGS: CuIn_1 - *x*_Ga_*x*_Se_2_; EDS: energy dispersive spectrum; fs: femtosecond; FWHM: full-width at half maximum; GBs: grain boundaries; HAZ: heat-affected zone; LPP: laser-produced plasmas; ns: nanosecond; PL: photoluminescence; PLD: pulsed laser deposition; SEM: scanning electron microscope; SLG: soda-lime glass; XRD: X-ray diffraction.

## Competing interests

The authors declare that they have no competing interests.

## Authors' contributions

SCC, DHH, and HJ carried out the thin film growth, XRD, SEM, and pump-probe analysis, and drafted the manuscript. YKL and FIL carried out the PL analysis. CHC participated in the design of the study. YLC, CWL, JYJ, KHW, and HCK conceived the study and organized the final version of the paper. All authors read and approved the final manuscript.
